# Supplementary figures and images for: The Effects of 6-Month Vitamin D Supplementation during the Non-Surgical Treatment of Periodontitis in Vitamin-D-Deficient Patients: A Randomized Double-Blind Placebo-Controlled Study
Source: Nutrients. 2020 Sep 25;12(10):2940. doi: 10.3390/nu12102940 (PMC7600700; doi:10.3390/nu12102940)

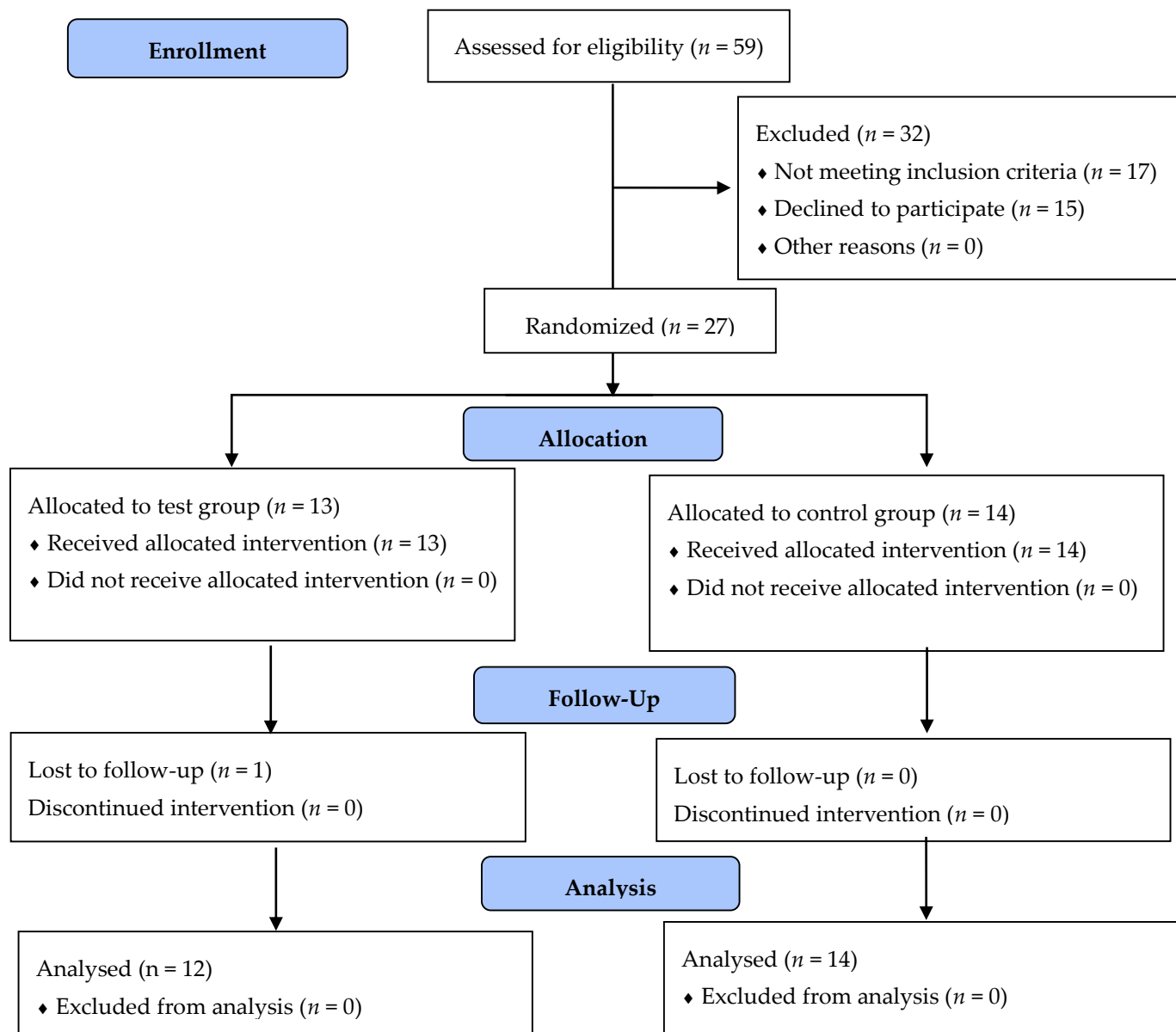

**Figure S1.** Consort flow chart.

Supplement: Supplementary file 1 [file nutrients-12-02940-s001.zip › Figure S1.pdf]
